# Precise Method for Measuring the Quadratic Electro-Optic Effect in Noncentrosymmetric Crystals in the Presence of Natural Birefringence

**DOI:** 10.3390/ma13183942

**Published:** 2020-09-06

**Authors:** Marek Izdebski, Rafał Ledzion, Włodzimierz Kucharczyk

**Affiliations:** Institute of Physics, Lodz University of Technology, Wólczańska 219, 90-924 Łódź, Poland; rafal.ledzion@p.lodz.pl (R.L.); wlodzimierz.kucharczyk@p.lodz.pl (W.K.)

**Keywords:** electro-optic effects, polarimetric method, natural birefringence, ADP crystal

## Abstract

The application of the improved dynamic polarimetric method for the measurement of the quadratic electro-optic effect in NH_4_H_2_PO_4_ (ADP) crystal with the light beam propagating perpendicularly to its optical axis is presented. This technique can be applied in noncetrosymmetric crystals in the presence of natural birefringence even when the fast and slow rays diverge slightly, causing them to only partially interfere. The method allows for minor errors in cutting and orientation of the crystal samples, resulting in deviations from configurations in which the crystal symmetry vetoes the linear electro-optic effect. The occurring contribution of the linear effect, if it is not too large, not only does not exclude the measurement of the quadratic effect, but increases its accuracy. The method does not require any prior compensation for the natural birefringence. Its sensitivity allows for quadratic electro-optic effect measurements in ferroelectrics in temperatures significantly different from the phase transition temperature or in paraelectric crystals, for which this effect is relatively small.

## 1. Introduction

Electro-optic effects in crystals are widely used in various technological devices [1,2,3]. These phenomena are also being studied to understand nonlinear interactions in crystals. The linear electro-optic effect (LEOE) is observed only in crystals that lack a center of inversion. The quadratic electro-optic effect (QEOE), on the other hand, is universal in the sense that it occurs in all media, irrespective of symmetry. Generally, when LEOE and QEOE occur together, the linear effect is the dominant one. So, to measure the quadratic effect, in crystals not possessing a center of symmetry, configurations theoretically forbidding the linear electro-optic response are considered [4,5]. However, even in configurations for which the symmetry vetoes a contribution of LEOE, experimental inaccuracies can lead to some response owing to the effect. Particularly troublesome are the measurements of small changes in birefringence induced by QEOE against the background of some LEOE response with simultaneous occurrence of natural birefringence. The fact that the natural birefringence is usually temperature-dependent makes measurements even more difficult. Therefore, sensitive techniques that, despite the presence of natural birefringence, are able to separate LEOE and QEOE are important.

A typical order of magnitude of LEOE is 10^−11^–10^−12^ mV^−1^. An extensive description of the measurement techniques used in the LEOE studies was presented by Aillerie et al. [6,7]. Some of the methods described therein, which use the initial natural birefringence compensation, can be used to measure LEOE in the presence of birefringence. Nevertheless, the fact that the QEOE is much smaller than LEOE makes the measurement of the former more complicated. QEOE in crystals that do not have a center of symmetry has been investigated mainly by polarimetric and interferometric means (see, for example, [1,2,3,8,9,10,11,12,13,14,15]). A technique based on the analysis of multiple reflections of polarized light on two faces of the investigated crystal has also been previously presented [16]. This method has been used to determine the QEOE coefficients in the ferroelectric phase of barium titanate at room temperature, where relatively large values of the QEOE coefficients, for example, 10^−17^ m^2^V^−2^, are observed. In paraelectric crystals and in ferroelectrics in temperatures significantly different from the phase transition temperature, the QEOE coefficients usually have much smaller values, for example, of the order of magnitude of 10^−20^ m^2^V^−2^ or lower.

Polarimetric methods are considered to be particularly simple in applications and, at the same time, precise. They are based on an analysis of changes in the light intensity propagating through an optical system consisting of a polarizer, sample, and analyzer. These changes result from the electric field-induced phase differences between interfering rays. The polarimetric technique is usually recognized as applicable in the measurement of induced birefringence in those cases where no natural birefringence occurs or the birefringence is compensated. Previously, we attempted to use a dynamic polarimetric technique to measure QEOE coefficients, which did not require any prior compensation for natural birefringence. We used the changing position of the modulator operating point on the characteristic of its transmission [10,14]. Recently, such a dynamic polarimetric method has been used to determine the sign of the QEOE coefficients [17].

The aim of this work is to present a description of the improved dynamic technique for QEOE measurements. The technique allows measurement of QEOE coefficients in noncentrosymmetric crystals even when only partial interference of the fast and slow beams occurs and LEOE manifests itself stronger than the quadratic one. We want to show that, instead of laboriously compensating for the temperature variable natural birefringence, the birefringence can be used as an aid in precise measurements. The method is illustrated by new results of measurements of the $n_{e}^{3}g_{3333}-n_{o}^{3}g_{1133}$ and $n_{e}^{3}g_{3311}-n_{o}^{3}g_{1111}$ coefficients of QEOE in NH_4_H_2_PO_4_ (ADP) crystals.

At room temperature, ADP is an optically uniaxial crystal belonging to the $\bar{4}2m$ point symmetry group. It is a member of the KH_2_PO_4_ (KDP) family, for which LEOE and optical second harmonic generation (SHG), the source of which is the second-order susceptibility, are well known. However, the phenomena resulting from third-order optical susceptibility are less well understood. We hope that the QEOE measurements can be used to reveal the contribution of the crystal lattice to the third-order susceptibility. The simple structure of the crystal can facilitate theoretical calculations using, for example, a bond polarizability approach [18]. QEOE in ADP also attracts attention owing to the spontaneous birefringence in its low-temperature antiferroelectric phase. Additionally, for ADP, one of the results obtained by the described method in the configuration resulting in the appearance of natural birefringence can be compared with that obtained earlier employing the interferometric technique with the light traveling along the crystal optical axis.

## 2. Materials and Methods

QEOE is often included in the analysis of the ferroelectric-paraelectric phase transition. In some ferroelectrics, for example, oxygen octahedra crystals, QEOE is analyzed for the relation between spontaneous birefringence and spontaneous polarization in the ferroelectric phase. Sometimes, LEOE is considered as fundamentally a quadratic one biased by the spontaneous polarization. The results of QEOE measurements are useful in analyzing the nature of the nonlinear susceptibilities of crystals. For example, the effect of deuteration on the electro-optic properties of deuterated KDP in the paraelectric phase was considered on the basis of the measured QEOE coefficients [10]. The contribution of PO_4_ groups to QEOE was also analyzed in these crystals [14]. In the case of ADP in the paraelectric phase, QEOE measurements were used to estimate the spontaneous antipolarization in its antiferroelectric phase [9,13]. It was shown that QEOE can be determined together with the electrostriction coefficients in the light passing through the crystal in the same experiment [12]. It is known that QEOE consists of the primary and electrostrictive-elasto-optic contributions. The primary contribution can be decomposed into terms owing to nonlinearities of purely electronic, purely lattice, and mixed electronic–lattice origin [18]. The purely lattice contribution results from an interaction of the applied electric field with the crystal lattice, which in turn changes the electronic polarizability and may be used to obtain the lattice contribution to the third-order nonlinear susceptibility. This nonlinear susceptibility is responsible for, among others, second-order Raman scattering and the second-order strain derivative of the electronic susceptibility. The electronic contribution is of the same origin as the third harmonic light generation. QEOE is also of interest from the point of view of technical applications. In devices based on the linear effect, the quadratic one is viewed as a disturbance. The fact that QEOE is related to various physical phenomena and can also influence practical applications makes its experimental research important. Such measurements require a sensitivity that allows for QEOE measurements in paraelectric noncentrosymmetric crystals or in ferroelectrics in the paraelectric phase.

The principle of the measurement method is presented below. We consider the total phase difference Γ*_t_* between the slow and fast waves in a linearly birefringent non-dichroic sample exposed to an external electric field **E** as composed of the part Γ*_c_* introduced by the sample independently of the field and the part Γ*_ind_*, which is induced in the sample

Γ*_t_* = Γ*_c_* + Γ*_ind_*, (1)

where

(2) $$\Gamma_{c}\;=\;\frac{2\pi L}{\lambda }\left|n_{o}{\;-\;n}_{e}\right|{\;\mathrm{and}\;\Gamma }_{ind}{\;=\;AE\;+\;BE}^{2}.$$

Here, *L* and λ are the crystal length and the light wavelength, respectively; and *n_o_* and *n_e_* are the ordinary and extraordinary refractive indices, respectively. The terms with coefficients *A* and *B* represent the phase differences due to LEOE and QEOE, respectively.

(3) $$A\;=\;\frac{2\pi Lr}{\lambda }\;\mathrm{and}\;B\;=\;\frac{2\pi Lg}{\lambda }.$$

According to the tensor matrices of QEOE (see, for example, [1,19]), in some crystal symmetries, for specific directions of the light and the electric field, the changes in Γ*_ind_* may be related to a combination of individual electro-optic coefficients. Such combinations are often called effective coefficients. In Equation (3), the linear and quadratic terms related to the electro-optic tensor components or their combinations are denoted as *r* and *g*, respectively.

We take into account the simplest polarimetric modulator consisting of a linear polarizer, an electro-optic crystal sample, and a linear analyzer. The response of such a system may be described by its working characteristic, that is, the dependence of the outgoing light intensity on Γ*_t_*. In the optimal measurement system, the azimuths of the polarizer and the fast wave in the sample form the angle ±45°. The light intensity transmitted by the modulator is given by

(4) $$I\;=\;\frac{I_{max}}{1\;+\;S}(1\;\pm \;S\cos\Gamma_{t}).$$

Here, *I_max_* is the maximum of the light intensity transmitted through the system and *S* is a dimensionless coefficient describing the partial interference of the fast and slow beam [20]. *S* takes values in the range 0…1 and describes an effective overlap of the beams. In Equation (4), the sign “+” corresponds to the case of parallel polarizers, while the sign “–” refers to crossed polarizers. When the light propagates perpendicularly to the optical axis of crystal, a natural birefringence appears, which is large relative to that electric-field-induced. Therefore, the electro-optic effect does not significantly affect the azimuth of the fast wave in the crystal sample. The use of trigonometric identity for the cosine of the sum of angles allows to express the emerging light intensity as

(5) $$I=\frac{I_{max}}{1\;+\;S}\left[1\;\pm \;S\left({\cos\Gamma }_{c}{\cos\Gamma }_{ind}-{\sin\Gamma }_{c}{\sin\Gamma }_{ind}\right)\right].$$

We consider a sinusoidal low-frequency modulating field *E* = *E*_0_ sin Ω*t*, hence

(6) $$\Gamma_{ind}{\;=\;AE}_{0}{\sin\Omega t+BE}_{0}^{2}\sin^{2}\Omega t.$$

Electro-optic measurements are usually carried out at small values of Γ*_ind_*; therefore, the expansions of the sine and cosine functions into power series give ${\sin\Gamma }_{ind}\approx \Gamma_{ind}$ and ${\cos\Gamma }_{ind}\approx 1-\Gamma_{ind}^{2}/2$. Using the transformation sin^2^Ω*t* to the form (1 – cos 2Ω*t*)/2 and taking into account that, in the expression for $\Gamma_{ind}^{2}$, the terms smaller than that resulting from the square of LEOE may be safely neglected, one rewrites Equation (5) as

(7) $$\begin{array}{c} I\;\approx \;\frac{I_{max}\;}{1\;+\;S}\{1\;\pm \;S[{\cos\Gamma }_{c}-\frac{1}{4}A^{2}E_{0}^{2}{\cos\Gamma }_{c}-\frac{1}{2}{BE}_{0}^{2}{\sin\Gamma }_{c}-{AE}_{0}{\sin\Gamma }_{c}\sin\Omega t\\ +\left(\;\frac{1}{4}A^{2}E_{0}^{2}{\cos\Gamma }_{c}+\frac{1}{2}{BE}_{0}^{2}{\sin\Gamma }_{c}\right)\cos2\Omega t]\}. \end{array}$$

Equation (7) shows that the light coming out of the modulator contains a non-modulated component *I*(0), a basic frequency component *I*(Ω), and a second harmonic component *I*(2Ω). Thus, it is convenient to use the signal voltage analysis in the light detection path. In our experiment, the voltage *U*(0) proportional to *I*(0) and the RMS voltages *U*(Ω) and *U*(2Ω) proportional to *I*(Ω) and *I*(2Ω), respectively, were recorded. The non-modulated component of the voltage is given by

(8) $$U\left(0\right)\;=\;\frac{U_{max}}{1\;+\;S}\left[1\;\pm \;S\left({\cos\Gamma }_{c}-\frac{1}{4}A^{2}E_{0}^{2}{\cos\Gamma }_{c}-\frac{1}{2}{BE}_{0}^{2}{\sin\Gamma }_{c}\right)\right].$$

Here, the terms proportional to $E_{0}^{2}$ are analogous in their origin to the phenomenon of optical rectification (OR) accompanying SHG. The LEOE contribution resulting from experimental inaccuracies leads to some response on the basic frequency Ω. One notes that its amplitude changes with changes in Γ*_c_*

(9) $$U(\Omega )\;=\;\frac{1}{\sqrt{2}}\frac{U_{max}}{1\;+\;S}S\left|A\right|E_{0}{\sin\Gamma }_{c}.$$

We will show that the component *U*(Ω) does not have to prevent measurements. In a sense, if it is controlled, it may be an indication that we actually measure QEOE and not the apparent effect that their source has in the linear effect and the nonlinearity of the modulator transmission characteristic. Moreover, some contribution of LEOE makes it easier to find points on the modulator characteristic where polarimetric measurements are the most accurate. These should be the points at which Γ*_c_* is close to π/2 + *k*π, where *k* = 0, 1, 2…, that is, close to the middle of the linear, most sensitive part of the modulator characteristic. Working near this point, for small changes Γ*_ind_*, as is in the case of electro-optic modulation, changes of light intensity are obtained that are practically a linear function of Γ*_ind_*. On the other hand, changes Γ*_ind_* near the points Γ*_c_* = *k*π cause changes in the light intensity proportional to the square of Γ*_ind_*. So, depending on the operating point, the changes in Γ*_ind_* owing to the linear effect can be observed at the modulator output at Ω or 2Ω frequency. Therefore, the *U*(2Ω) component has its source in both the linear and the quadratic effect

(10) $$U(2\Omega )\;=\;\frac{1}{\sqrt{2}}\frac{U_{max}}{1\;+\;S}S\left|\frac{1}{4}A^{2}{\cos\Gamma }_{c}+\frac{1}{2}{B\sin\Gamma }_{c}\right|E_{0}^{2}.$$

The contributions of these effects can usually be clearly separated. This can be obtained using the fact that light modulation at the frequency of 2Ω resulting from the existence of the real QEOE coefficient *g* changes like sin Γ*_c_*. In turn, the 2Ω response owing to the linear effect *r* changes in proportion to cos Γ*_c_*.

**Figure 1:**
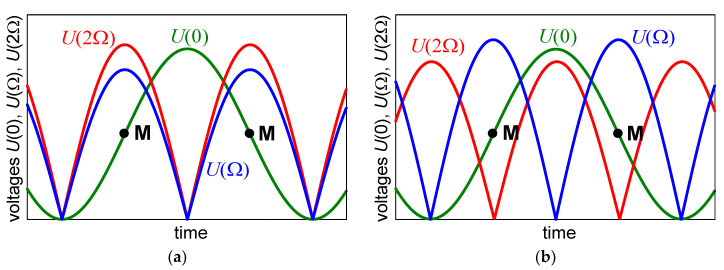
An example of theoretical time dependencies of the voltages *U*(0), *U*(Ω), and *U*(2Ω), as expected from Equations (8), (9), and (10), respectively. The phase shift Γ*_c_* increases linearly with time. Points **M** indicate the middle of the most sensitive linear part of the transmission characteristic of the modulator given by Equation (5), where Γ*_c_* = π/2 + *k*π. (**a**) A case of well enough cut and oriented sample; (**b**) an example of false results that can be obtained in the case of an insufficiently precisely cut or poorly oriented sample in a noncentrosymmetric crystal.

Equations (8)–(10) allow to expect the dependencies of *U*(0), *U*(Ω), and *U*(2Ω) on Γ*_c_*, as shown in Figure 1.

In our measurements, the variable phase Γ*_c_*, which allowed a smooth shift of the operating point through the transmission characteristics of the modulator, was ensured by changes in the sample temperature. The temperature affected the refractive indices, and also changed the sample size, causing changes in the light path *L*.

Information on the magnitude of the QEOE coefficient can be obtained from the 2Ω response amplitude. The most suitable moments for measuring the quadratic effect correspond to these values Γ*_c_* when the unwanted contribution of the linear effect to *U*(2Ω) disappears, that is, when cos Γ*_c_* = 0, while the contribution of the quadratic effect becomes maximum. The corresponding *U*(2Ω) can be expressed as

(11) $${U(2\Omega )|}_{{\cos\Gamma }_{s}=\;0}\;=\;\frac{1}{2\sqrt{2}}\frac{U_{max}}{1\;+\;S}S\left|B\right|E_{0}^{2}.$$

To determine the moments when cos Γ*_c_* = 0, one can use the fact that the component *U*(0) given by Equation (8) then passes through an approximately average value between its minimum and maximum, while the component *U*(Ω) given by Equation (9) reaches a maximum. The exact determination of the moment when *U*(Ω) is maximal is experimentally simple, which shows that a certain contribution of LEOE can actually facilitate and increase the accuracy of the QEOE measurements.

The factor (*U*_max_ *S*) ⁄ (1 + *S*) appearing in Equation (11) can be found using the amplitude of changes in the *U*(0) component given by Equation (8) for the variable phase Γ*_c_*:

(12) $$\Delta U(0)=\frac{1}{2}\left[U_{\max}\left(0\right)-U_{\min}\left(0\right)\right]=\frac{U_{max}}{1\;+\;S}S\sqrt{{\left(1-\frac{1}{4}A^{2}E_{0}^{2}\right)}^{2}+{\left(\frac{1}{2}{BE}_{0}^{2}\right)}^{2}}.$$

The most accurate way to determine *U*_max_(0) and *U*_min_(0) values is to find the moments when *U*(Ω) reaches the minimum, which is again relatively simple experimentally. Measurements of QEOE are usually carried out at small depths of light intensity modulation. For example, in our experiments, the depth modulation factor *U*(2Ω)/*U*(0) was of the order of 10^−3^ or lower. Thus, the contribution of the terms $A^{2}E_{0}^{2}$ and ${BE}_{0}^{2}$ to the value of Δ*U*(0) does not exceed about 10^−3^·Δ*U*(0) and may be neglected in Formula (12). Using Equations (11) and (12), and expanding the term *B* as defined in Formula (3), the quadratic electro-optic coefficient *g* can be expressed as

(13) $$\left|g\right|=\frac{\sqrt{2}\lambda {U(2\Omega )|}_{{\cos\Gamma }_{c}=\;0}}{{\pi \Delta U(0)\;LE}_{0}^{2}},$$

where the *g* values correspond to those temperatures that are related to individual **M** points.

As already mentioned, despite the fact that the measurements are carried out in a configuration that theoretically excludes the participation of the linear effect, in practice, this effect can only be significantly reduced, and not completely excluded. If the tested sample is not cut or oriented precisely enough and the contribution of the linear effect becomes too large, the measurement of the actual quadratic effect becomes impossible. Moreover, misinterpretation of changes in *U*(2Ω) may result in an incorrect, larger than in reality value of QEOE. An example of the nature of the readings that can be obtained in such a situation is shown in Figure 1b.

Comparing the drawings shown in Figure 1a,b, it can be seen that, when the maximum *U*(2Ω) is in a vicinity of the minimum *U*(Ω) and, at the same time, with the minimum or maximum *U*(0), the 2Ω component is mainly or entirely attributable to LEOE and the nonlinearity of the modulator characteristic. Therefore, it cannot be a source of QEOE information. Such results as those shown in Figure 1b should be rejected and the sample alignment corrected. Our experiments with KDP type crystals show that the sample cutting error of 2 degrees can be compensated by proper sample orientation. In this, instead of orienting the crystal face perpendicularly to the light beam, it is necessary to find a position where *U*(Ω) is minimal. If this fails, the use of a better cut sample or a sample of better crystallographic quality should be considered.

## 3. Results

We performed measurements for ADP crystals. The following two directions of light and electric field were considered. For sample A,

**σ** = (1, 0, 0), **Ε** = (0, 0, *E*), (14)

and for sample B,

**σ** = (0, 1, 0), **Ε** = (*E*, 0, 0). (15)

In both configurations, the light propagated perpendicularly to the crystal optical axis and a natural birefringence appeared. According to the literature [1,19], in configurations (14) and (15), the induced birefringence Γ*_ind_* is given by the formula

(16) $$\Gamma_{ind}\;=\frac{\pi L}{\lambda }\left(n_{e}^{3}g_{3333}-n_{o}^{3}g_{1133}\right)E^{2},$$

and

(17) $$\Gamma_{ind}\;=\frac{\pi L}{\lambda }\left(n_{e}^{3}g_{3311}-n_{o}^{3}g_{1111}\right)E^{2},$$

respectively.

**Figure 2:**
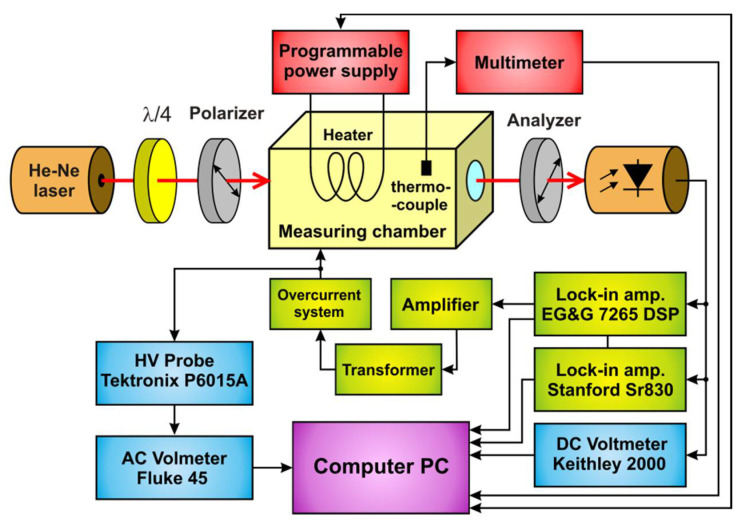
Diagram of the optical and electronic components employed in the measurements. The He-Ne laser Melles Griot 05-LPH-171, quarter-wave plate Melles Griot 02WRM001, and two Melles Griot 03FPI025 polaroid type polarizers were used. The laser used had linear polarization. An additional quarter-wave plate made it possible to obtain circular polarization and become independent of the laser position. Temperature was measured using a copper-constantan thermocouple.

The experimental setup used in our measurements is presented in Figure 2. The crystal samples were cut in the form of right parallelepipeds. Their edges were parallel to the crystallographic axes *X*, *Y*, *Z*, with the *Z* axis along the optical axis. The dimensions of the samples were 24.05 × 23.60 × 4.26 mm^3^ and 5.01 × 50.27 × 51.22 mm^3^ (*X* × *Y* × *Z*) for samples A and B, respectively. To apply the electric field, the samples were coated with a silver conducting paint on their two faces. Modulating voltages with the frequency of 417 Hz and RMS values of 2553 V and 1931 V were applied for samples A and B, respectively. The frequency was chosen to ensure a relative low noise in our experimental system. In our case, the noise level in the detection path decreased with the increasing frequency of the modulating electric field. At the same time, the increase in frequency caused the reduction of the transformer ratio. A compromise between these effects was the selected frequency of 417 Hz, which also ensured the absence of harmonics in the mains (50 Hz). The frequency is over 100 times lower than the expected mechanical resonance frequency for the samples tested. Different samples loaded the transformer differently; therefore, the actual voltage applied to the crystals was measured directly on the samples. To suppress the effect of multiple reflections and to protect the hygroscopic crystals against moisture, the samples were immersed in a cuvette with a methyl silicone oil whose QEOE was previously determined. It was found that the contribution of QEOE in the oil owing to a fringing electric field may be neglected. Samples A and B, resting freely on their supports, were mechanically free. The voltage was applied to the crystals with copper wires 1 × 10^−4^ meters in diameter. The small diameter of these lines reduced the possibility of introducing mechanical stress. It also protected the samples from vibration in high voltage cables. The frequency of the modulating field was much lower than the frequency of mechanical resonances of the samples, thus mechanically free values of the QEOE coefficients were determined.

**Figure 3:**
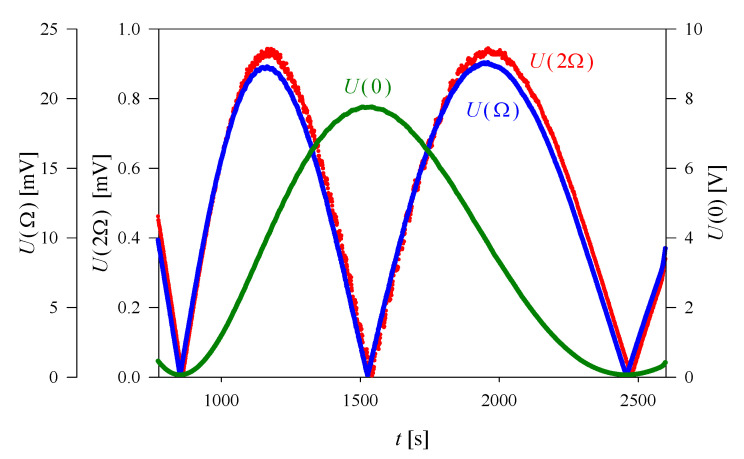
An example of the results obtained in quadratic electro-optic effect (QEOE) measurements in NH_4_H_2_PO_4_ (ADP) for sample A in the configuration given by Equation (14). The sample was heated, and the experimental data shown in the graph correspond to the temperature range 295.69–296.49 K.

**Figure 4:**
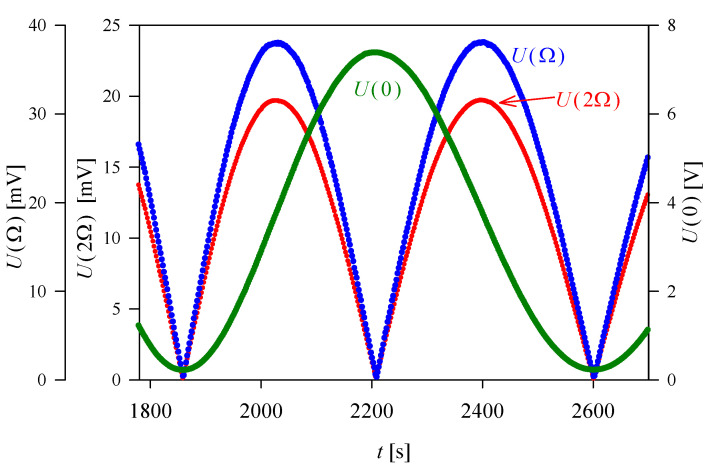
An example of the results obtained in QEOE measurements in ADP for sample B in the configuration given by Equation (15). The sample was heated, and the experimental data shown in the graph correspond to the temperature range 295.50–295.80 K.

The corresponding experimental results obtained for *U*(0), *U*(Ω), and *U*(2Ω) are shown in Figure 3 and Figure 4 for sample A and B, respectively. The application of Equation (13) gave the following values of the QEOE coefficients:

(18) $$\left|n_{e}^{3}g_{3333}-n_{o}^{3}g_{1133}\right|\;=\;(0.81\;\pm \;0.04)\;\times \;10^{-20}\;m^{2}V^{-2}\;\mathrm{at}\;295\;K,$$

(19) $$\left|n_{e}^{3}g_{3311}-n_{o}^{3}g_{1111}\right|\;=\;(20.89\;\pm \;0.14)\;\times \;10^{-20}\;m^{2}V^{-2}\;\mathrm{at}\;295\;K.$$

## 4. Discussion

In both of the above configurations, QEOE in ADP has not yet been measured. Nevertheless, the individual *g*_3311_ and *g*_1111_ coefficients in ADP have previously been measured with an actively stabilized Michelson interferometer employing a compensation method in a configuration excluding natural birefringence when the light propagated along the optical axis [11].

The result $\left|n_{e}^{3}g_{3311}-n_{o}^{3}g_{1111}\right|$ = (20.89 ± 0.14) × 10^−20^ m^2^V^−2^ at 295 K obtained for sample B in the configuration in Equation (15) agrees with the value $\left|n_{e}^{3}g_{3311}-n_{o}^{3}g_{1111}\right|$ = (21.4 ± 4.6) × 10^−20^ m^2^V^−2^ [11], which can be obtained with the help of the coefficients $g_{3311}$ = (–1.4 ± 0.9) × 10^−20^ m^2^V^−2^ and $g_{1111}$ = (−7.4 ± 1.0) × 10^−20^ m^2^V^−2^ determined at room temperature by interferometric means [11], and the literature values of the refractive indices *n_o_* and *n_e_* [21]. According to our knowledge, the effective coefficient $n_{e}^{3}g_{3333}-n_{o}^{3}g_{1133}$ in ADP has not been previously reported.

The method discussed in the article requires a noticeable temperature dependence of the phase difference introduced by the sample. If this condition is not met, an additional crystal can be used to shift the operating point on the modulator characteristic. An example of such a measurement is shown in [17], where the configuration with the light being sent along the optical axis of uniaxial crystal was taken into account. This approach (with or without an additional crystal) cannot be used for samples exhibiting significant dichroism or natural optical activity. In our opinion, for dichroic crystals, by slightly complicating it, the method can be extended in the future.

## 5. Conclusions

The improved dynamic polarimetric method enables precise measurements of the QEOE coefficients in noncetrosymmetric crystals against the background of natural birefringence. It can be applied even when there is only partial interference of fast and slow rays. Although the method should generally be used in those configurations where the symmetry of the crystal excludes the linear effect, in practice, it allows for minor exceptions to this rule. The appearing contribution of the linear effect, if it is not too large, increases the precision of the measurement. The possibility of observing the contribution of the linear effect ensures that it does not uncontrollably increase the result of the QEOE measurement. The approach also enables the measurement in the case of slightly incorrectly cut samples, when the LEOE attenuation condition by the crystal symmetry is satisfied with not completely perpendicular incidence of the light ray on the entrance face of the crystal. The method does not require any prior compensation for the natural birefringence. Its sensitivity allows for QEOE measurements in paraelectric crystals and ferroelectrics at temperatures significantly different from the phase transition temperature, where the effect is relatively small. In combination with the procedure described earlier [17], the technique allows to determine the sign of the measured QEOE coefficients. This approach can also be used in the usually relatively easier LEOE measurements.
